# Datasets on extinction coefficients for free space optical link survey and optimization

**DOI:** 10.1016/j.dib.2018.12.012

**Published:** 2018-12-08

**Authors:** Cheikh Amadou Bamba Dath, Aliou Niane, Ndeye Arame Boye Faye, Modou Mbaye

**Affiliations:** Laboratoire Atomes Lasers, Faculté des Sciences et Techniques, Université Cheikh Anta Diop de Dakar (UCAD), Sénégal

## Abstract

Based on visibilities data recorded from 2004 to 2013, the minimum, maximum and mean values of extinction coefficients were determined and analyzed in a monthly basis, a yearly basis and also for the whole period of observation.

The extinction coefficients data are obtained for the 1330 and 1550 nm optical wavelengths and may be used inter alia for range and availability analyses of optical link for different weather conditions. The data are collected in the region of Dakar, but approach and model of investigation can be reproduced for other regions in Sahel, and in the World, for optical metrology and allied fields of study.

**Specifications table**

**Table:** 

Subject area	*Physics, Chemistry,*
More specific subject area	*Free Optical communication, Optical signal metrology in atmosphere*
Type of data	*Table (in word files), graphs, figures*
How data was acquired	*Survey of visibilities data of Word meteorological station in Dakar, via free web portal*
Data format	*Filtered, analyzed, and processed for 1330 nm and 1550 nm*
Experimental factors	*Data of visibility are computed by using Beer Lambert Law of transmittance and with combination with the Kruse Model, in regard to obtain extinctions coefficients at 1330 nm and 1550 nm*
Experimental features	*Extinctions value are obtained by using and analyzing visibility data, measured by transmissiometers or Diffusometer with error measurement of less than 1%. So, error induced in propagation can be neglected for free space optical communications purposes.*
Data source location	*City of Dakar, in Senegal*
Data accessibility	*Data is provided with this current article*

**Value of the data**

• The present data will be of great usefulness and utility for the determination of optical signal range in atmosphere;
• The data will help to analyze the range and availabity of free space optical systems over the time, as in a monthly, seasonal or yearly basis
• The data can be used for optical communication power budget modelling, forecasting and planning.
• The data can be used as support for comparison and survey of feasibility studies of optical applications in other regions, by professional and scientists.

## 1 Data

**Fig. 1:**
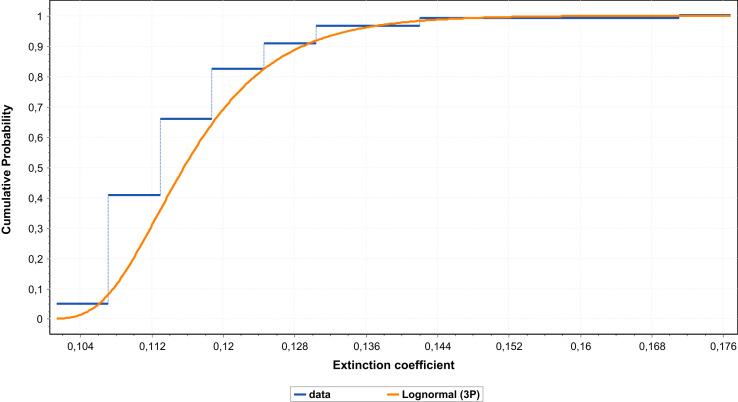
Cumulative distribution function of the lognormal (3P) of the data at 1550 nm.

**Fig. 2:**
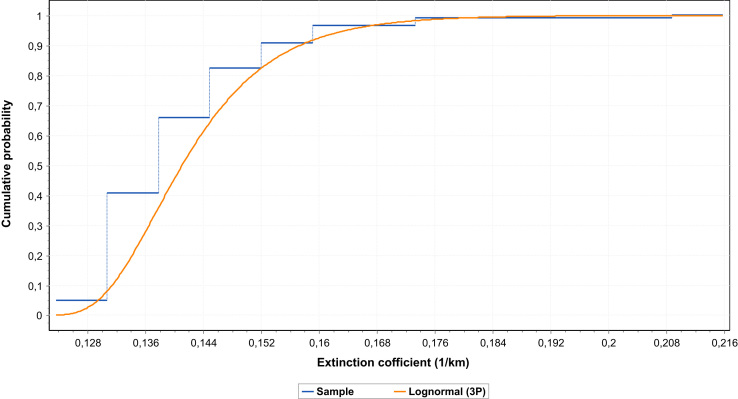
Cumulative distribution function of the lognormal (3P) of the data at 1330 nm.

The datasets used in this work contain extinction coefficients for Dakar weather, from January 1, 2004 to December 31, 2013. The datasets are minimum, maximum and mean values of extinction coefficients at 1330 and 1550 nm, presented respectively in Table 1, Table 2, Table 3. Also, lognormal approximations of monthly mean extinction coefficients presented in Table 4 and corresponding to 120 values of the ten years of observation are presented within two figures, noted Fig. 1, Fig. 2. The extinction coefficients were estimated at both 1330 and 1550 nm by using a method called Kruse model. The data serve for survey, planning and optimization of free space optical communications systems and in general for optical metrology and spectroscopy in the near infra-red window.

The computed data, the modeling approach and the related precisions are described in this paper.

**Table 1:** Extinction coefficient computed for monthly mean of minimum visibility from 2004–2013.

Month	λ = 1550	λ = 1330
Jan	0.330428811	0.382461926
Feb	0.266951974	0.311463881
Mar	0.403887066	0.464124991
Apr	0.14553316	0.177576721
May	0.155785113	0.190085955
Jun	0.133151412	0.162468754
July	0.121538445	0.148298839
Aug	0.129754692	0.158324143
Sept	0.13025311	0.158932303
Oct	0.139928031	0.170737453
Nov	0.144911223	0.176817847
Dec	0.219545988	0.258104977

**Table 2:** Extinction coefficient computed for monthly mean of maximum visibility from 2004–2013.

Month	λ = 1550	λ = 1330
Jan	0.107648337	0.1313504
Feb	0.104122496	0.127048238
Mar	0.104122496	0.127048238
Apr	0.104016031	0.126918332
May	0.101626053	0.124002126
Jun	0.100224314	0.122291752
July	0.093413846	0.113981752
Aug	0.097533728	0.119008752
Sept	0.098477908	0.120160821
Oct	0.098382668	0.120044611
Nov	0.104550543	0.127570533
Dec	0.106632787	0.130111246

**Table 3:** Extinction coefficient computed for monthly mean of mean visibility from 2004–2013.

Month	λ= 1550	λ= 1330
Jan	0.133307881	0.16266
Feb	0.124888337	0.152386
Mar	0.125368959	0.152973
Apr	0.116436699	0.142074
May	0.114601447	0.139834
Jun	0.111963016	0.136615
July	0.109336049	0.13341
Aug	0.108973761	0.132968
Sept	0.110712607	0.135089
Oct	0.112704877	0.13752
Nov	0.115631937	0.141092
Dec	0.121295352	0.148002

**Table 4:** Extinction coefficient at 1550 nm and 1330 nm.

**Extinction coefficient at** λ **= 1550 nm**			**Extinction coefficient at** λ= **1330 nm**		
0.124792891	0.118644059	0.113030745	0.152269862	0.144767177	0.13791792
0.131995576	0.111706771	0.112747873	0.161058439	0.136302434	0.137572766
0.14588105	0.10635941	0.119258699	0.178001225	0.129777687	0.145517149
0.110325442	0.10719098	0.114883691	0.134616963	0.130792353	0.140178849
0.110204967	0.113210498	0.125290411	0.134469961	0.138137252	0.152876928
0.111198685	0.10855618	0.125203286	0.135682476	0.132458143	0.152770619
0.108451674	0.115373598	0.11434225	0.132330627	0.140776624	0.139518194
0.103439806	0.118243579	0.112857821	0.126215243	0.144278519	0.137706923
0.103150424	0.142704175	0.111511952	0.125862145	0.174124864	0.136064719
0.111380701	0.122090688	0.112613699	0.13590457	0.148972687	0.137409049
0.115013709	0.120757336	0.111748984	0.140337495	0.147345757	0.136353941
0.128769203	0.12276743	0.113030745	0.157121681	0.149798434	0.13791792
0.176868164	0.110737735	0.110814456	0.2158111	0.135120035	0.135213647
0.128189753	0.109659684	0.114549802	0.156414648	0.133804618	0.139771445
0.124400691	0.108819665	0.112447641	0.151791309	0.132779643	0.137206429
0.122514305	0.111433165	0.128245507	0.149489576	0.135968584	0.156482677
0.118778017	0.10997586	0.135216281	0.14493063	0.134190409	0.164988281
0.110854668	0.11882283	0.131349133	0.135262713	0.14498531	0.160269662
0.110148764	0.116526541	0.133286432	0.134401383	0.142183423	0.162633517
0.10635941	0.124893397	0.11468734	0.129777687	0.152392498	0.139939265
0.111952641	0.127829735	0.114011497	0.136602439	0.155975361	0.139114615
0.113929139	0.119629368	0.112282197	0.139014124	0.145969432	0.137004557
0.116171639	0.120456771	0.111354488	0.141750378	0.146979013	0.135872585
0.122230931	0.123957306	0.111275802	0.149143809	0.151250298	0.135776573
0.120745009	0.117058601	0.113493166	0.147330716	0.142832633	0.138482158
0.118440998	0.116215966	0.11202686	0.144519406	0.141804466	0.136692999
0.13283739	0.111236622	0.113619927	0.162085604	0.135728766	0.138636829
0.115033217	0.107703491	0.117582358	0.140361299	0.131417708	0.14347171
0.112146408	0.110693874	0.122073253	0.13683887	0.135066516	0.148951414
0.108916135	0.112066722	0.13042009	0.132897354	0.136741638	0.159136062
0.101335401	0.123356058	0.112707525	0.123647489	0.150516667	0.137523533
0.105153682	0.118199339	0.115731138	0.128306482	0.144224539	0.141212888
0.108029346	0.127933425	0.112655227	0.131815311	0.156101882	0.137459721
0.110923567	0.12141412	0.11378934	0.135346783	0.148147152	0.138843544
0.111829571	0.130744437	0.112988939	0.136452271	0.159531824	0.13786691
0.119182132	0.117649535	0.113970239	0.145423723	0.143553678	0.139064273
0.145998086	0.121477624	0.113662201	0.17814403	0.148224638	0.138688411
0.121986009	0.112738501	0.112613699	0.14884496	0.137561331	0.137409049
0.125141216	0.111947343	0.113619927	0.152694883	0.136595975	0.138636829
0.110733999	0.111236622	0.122325734	0.135115475	0.135728766	0.149259485

## 2 Experimental design, materials and methods

The extinction coefficients data are derived from visibility measurements, recorded for a period of ten years, from 2004 to 2013. So extinction coefficient depends on absorption and scattering of the propagating optical signal in the earth׳s atmosphere [1], [2], [3], [4], [5].

Several following equations are used to obtain the extinction coefficient; before all, we have the atmospheric transmittance which is expressed by the beer-Lambert׳s law [1], [2], [4]:

(1) $$\tau \left(L\right)=\frac{P\left(L\right)}{P\left(0\right)}=e^{\left(-\gamma \left(\lambda \right)L\right)}$$

And γ(λ)is the extinction coefficient which is approximately taken to be equal to that produced only by the aerosols and is expressed as [3], [4], [5], [6]:

(2) $$ɣ(\lambda )=\frac{3.912}{V}({\frac{\lambda_{\mathrm{nm}}}{550})}^{-q}$$

Coefficient q has been established within many experimental studies and may take the values below, subject to the visibility range, according to KRUSE [1], [4], [6], [7]

(3) $$q=\{\begin{array}{cc} 1.6\; & if\;V\;>50\;km\\ 1.3\; & if\;6\;km<V<50\;km\\ 0.585V^{1/3} & \;if\;V<6\;km \end{array}\;$$

*V* is the atmospheric visibility which is measured by transmissiometers or diffusometers sensors.

Visibility and extinction are here phenomenon varying with time and period [3], [4], [7], [8].

The datasets of extinction coefficients at 1330 and 1550 nm 1550 nm presented the tables (Table 1, Table 2, Table 3, Table 4), are obtained by adopting that Kruse model.

The data of the Table 4 represent the average extinction coefficient for each month, over the period from 2004 to 2013, corresponding to the 120 monthly values over the ten years of observation [8].

### 2.1 Lognormal approximation and precision of data analyses for 1550 nm

A lognormal approximation of extinction coefficients data is performed at 1550 nm. A descriptive statistical analysis of the 120 values of extinction coefficient at the wavelength of 1550 nm is resumed in the Table 5.

**Table 5:** Descriptive statistic parameters at 1550 nm.

Statistic	Value	Percentile	Value
Sample size	120	Min	0.10134
Range	0.07553	5%	0.1064
Mean	0.11739	10%	0.10883
Variance	9.8911E-5	25% (Q1)	0.11139
Std. deviation	0.00995	50% (Median)	0.11399
Coef. of variation	0.08472	75% (Q3)	0.12205
Std. Error	9.0789E-4	90%	0.12872
Skewness	2.4257	95%	0.13326
Excess kurtosis	10.488	Max	0.17687

The data are analyzed again, in regard to see what kind of distribution, they may follow, and to provide a tool which is easy to use by professionals and other non-technical people. The lognormal approximation proposed is fitted with very good precision [8].

The values of the parameters of the lognormal approximation (3P) at 1550 nm are: *σ* = 0.40726; *µ* = −3.9384;.

*ɣ* = 0.09615 and the number of bins used is 13; the cumulative distribution is fitted in the Fig. 1.

### 2.2 Lognormal approximation and precision of data analyses for 1330 nm

A lognormal approximation of extinction coefficients data at 1330 nm is also performed; a descriptive statistical analysis of data is resumed in the Table 6.

**Table 6:** Descriptive statistic parameters at 1330 nm.

Statistic	Value	Percentile	Value
Sample size	120	Min	0.12365
Range	0.09216	5%	0.12983
Mean	0.14324	10%	0.13279
Variance	1.4726E-4	25% (Q1)	0.13592
Std. deviation	0.01214	50% (Median)	0.13909
Coef. of variation	0.08472	75% (Q3)	0.14892
Std. Error	0.00111	90%	0.15706
Skewnes Excess Kurtosis	2.4257	95%	0.16261
Statistic	10.488	Max	0.21581

The values of the parameters of the lognormal approximation (3P) at 1330 nm are: *σ* = 0.40726;.

*µ* = −3.7394; *γ* = 0.11732 [8]. The cumulative distribution function is presented in Fig. 2.

The goodness of the lognormal fit at the different wavelengths (1550 and 1330 nm) are estimated with very high precision (Table 7).

**Table 7:** Goodness of Lognormal (3 P) fit for data at 1330 nm and 1550 nm.

**Items**	**Goodness of Lognormal (3P) fit for data at 1330 nm**					**Goodness of Lognormal (3P) fit for data at 1550 nm**				
**Kolmogorov-Smirnov**										
Sample size	120					120				
Statistic	0.09278					0.09278				
*P*-Value	0.23774					0.23773				
Rank	10					9				
*α* (significance level)	0.2	0.1	0.05	0.02	0.01	0.2	0.1	0.05	0.02	0.01
Critical value	0.09795	0.11164	0.12397	0.13857	0.14871	0.09795	0.11164	0.12397	0.13857	0.14871
Reject?	No	No	No	No	No	No	No	No	No	No

The goodness of lognormal (3P) fit for data at 1330 nm and 1550 nm are performed by using the method of Kolmogorov-Smirnov.
