# Supplementary material for: Datasets on extinction coefficients for free space optical link survey and optimization
Source: Data Brief. 2018 Dec 8;22:464–70. doi: 10.1016/j.dib.2018.12.012 (PMC6312795; doi:10.1016/j.dib.2018.12.012)
Supplement: Supplementary file 1 — Supplementary material [file mmc1.docx]

Conflict of Interest Form

*Data article*

**Title: Datasets on extinction coefficients for free space optical link survey and optimization**

**First Author:** **Cheikh Amadou Bamba DathAffiliations: Laboratoire Atomes Lasers, Faculte des Sciences et Techniques, Université Cheikh Anta Diop de Dakar (UCAD)**

**Contact email:** [**bambadath@yahoo.fr**](mailto:bambadath@yahoo.fr)**;** [**cheikhamadou.dath@ucad.edu.sn**](mailto:cheikhamadou.dath@ucad.edu.sn)

**Acknowledgements of Autor**

**Dr Cheikh Amadou Bamba Dath, First author of the article declare that no charge is associated in use of material and document related to the article. The article is free of use and there are no lucrative behavior foreseen in that article.**

**Cheikh Amadou Bamba Dath**

**22 november 2018**
